# High Order Linguistic Features Such as Ambiguity Processing as Relevant Diagnostic Markers for Schizophrenia

**DOI:** 10.1155/2012/825050

**Published:** 2012-12-11

**Authors:** Daniel Ketteler, Anastasia Theodoridou, Simon Ketteler, Matthias Jäger

**Affiliations:** ^1^ZADZ Zurich, Dufourstrasse 161, 8008 Zürich, Switzerland; ^2^Research Group Clinical and Experimental Psychopathology, Department of Social and General Psychiatry, Psychiatric University Hospital Zurich, Lenggstraße 31, 8008 Zurich, Switzerland; ^3^Department of Neurology, RWTH Aachen University, Pauwelsstraße 30, 52074 Aachen, Germany

## Abstract

Due to the deficits of schizophrenic patients regarding the understanding of vague meanings (D. Ketteler and S. Ketteler (2010)) we develop a special test battery called HOLF (high order linguistic function test), which should be able to detect subtle linguistic performance deficits in schizophrenic patients. HOLF was presented to 40 schizophrenic patients and controls, focussing on linguistic features such as ambiguity, synonymy, hypero-/hyponymy, antinomy, and adages. Using the HOLF test battery we found that schizophrenic patients showed significant difficulties in discriminating ambiguities, hypero- and hyponymy, or synonymy compared to healthy controls. Antonyms and adages showed less significant results in comparing both groups. The more difficult a linguistic task was, the more confusion was measured in the schizophrenic group while healthy controls did not show significant problems in processing high order language tasks.

## 1. Introduction

Regarding the history of diagnostic classification of schizophrenia, diagnostic tools and catalogues focussed on different symptoms to describe a complex syndrome called schizophrenia. On the one hand, Bleuler [1] had concentrated on the phenomenon of loosening of association to classify and explain schizophrenian symptoms. According to Bleuler, language-based “loosening of association” is pathognomonic for the so-called “schizophrenic symptoms complex.” On the other hand, Schneider [2] drew attention to the significance of “core” or “first rank” symptoms first outlined by Kraepelin (specific types of hallucination and thought disorder [3]). To overcome the at least obscure relationship between thought and association disorder of Bleuler's approach, Andreasen [4, 5] shifted the focus of investigation from “thought” to the more objectively measurable “language behaviour.” Language impairment indeed seems to be one of the “core” phenomenological characteristics of patients with schizophrenia [6, 7]. It seems to be clear that there are deficits in the neural organisation of language in schizophrenic patients [6, 8].

There is only a small number of studies focussing on high order linguistic features and particularly on the phenomenon of ambiguity. Salisbury et al. [9] described a model of initial hyperpriming and subsequent decay of information by using ERP data investigating patients with schizophrenia. Using event related brain potentials and an ambiguity processing paradigm, Salisbury [10] found that schizophrenia patients showed the largest N400 effect to subordinate associates, with less activity to dominant meaning associates and unrelated words. These findings suggest a neural correlate for the difficulties in suppressing correct word alternatives. 

Several other aspects of language comprehension and production have been found to be abnormal in patients with schizophrenia: comprehension, attention, semantic organisation, reference failures, paucity of speech, or fluency [11]. Covington et al. [12] discussed that thought disorder might reflect a disruption of executive function and pragmatics. Although normal with regard to segmental phonology and morphological organisation [13], there are obvious word-finding difficulties in patients with schizophrenia [4, 14]. Disturbed language production often includes deictic terms with no clear referents and verbs which lead to a vague and ambiguous discourse. Difficulties in dealing with nonliteral expressions were found by Corcoran and Frith [15] and Langdon et al. [16]. Particularly vague and ambiguous terms seem to irritate patients with schizophrenia. Some studies investigating comprehension deficits attributed reduced comprehension to deficient working memory [17], and some found deficits in semantic processing [18]. 

Beside behavioural difficulties in solving high order language tasks there are neuroanatomic and neurofunctional changes, especially regarding language pathways, in patients with schizophrenia [19–25]. 

Regarding the aetiology of schizophrenia from a neurolinguistic point of view, there might be hemispheric interaction difficulties particularly in processing high order linguistic features such as semantic ambiguities. Besides cortical abnormalities, the subcortical role of language processing was underestimated for a long time. 

Deficits of schizophrenic patients in resolving vague meaning probably enable test batteries for the early detection of psychosis and might lead to a better understanding in the aetiology of schizophrenia in general. Ceccherini-Nelli and Crow [26] used a psychometric test (CLANG) to evaluate language disturbances and found that language symptoms like semantic/phonemic paraphasias or poverty of speech were superior to nuclear symptoms in discriminating ICD-10 schizophrenia from other psychoses. CLANG was originally developed by Chen et al. [27]. This task highly depends on the experience of the examiner and is not appropriate to detect subtle language symptoms. We argue that the CLANG test focuses on symptoms that are more commonly seen during episodes of acute illness. We tried to develop a more elaborated test battery which is able to detect more subtle language deficits in well-medicated patients with low symptom load. The so-called HOLF (high order linguistic function test) was presented to 40 schizophrenic patients and controls focussing on linguistic features. HOLF is a pilot test battery created by our group containing different high order language features such as antonyms, homonyms, synonyms versus hyponyms, and adages. This experiment is the first attempt to introduce our test design to the scientific community. It has not been published before; however, there was one study by Ketteler et al. [24] regarding the homonym part using fMRI with healthy individuals.

## 2. Method and Subjects

40 schizophrenic patients (27 male, 13 female) with a mean age of 31,54 (sd = 10,834) and 40 controls (27 male, 13 female) with a mean age of 32,48 (sd = 9,081) participated in the study.All participants were monolingual German native speakers and had no history of neurological disorder or a history of head trauma. Schizophrenia was diagnosed by an experienced clinician using the ICD-10 criteria. To determine the symptom load of the schizophrenic patients we used PANSS [26] as a well-established diagnostic instrument. For the language symptoms CLANG [28] was rated by an experienced examiner. Furthermore, age, gender, and CGI (Clinical Global Impression Score [27]) were registered in all participants and number of previous hospitalizations in the clinical subsample.

## 3. Experimental Design and Procedure

A linguistic task (HOLF) was presented to 40 patients with schizophrenia and 40 controls. The first task was a warm-up task consisting of 20 antonym relation pairs, while half of them were distractors. The second task represented other, mixed high order linguistic features, including synonymy, homonymy, and hyperonomy versus hyponomy. Each item group included 20 items while 10 of them were correct, and 10 of them were distractors. Individuals were instructed by the examiner: “Please mark if the first words correlate with the last word in the line” (for details see HOLF test in German language attached in supplementary material available online at doi:10.1155/2012/825050).

Example (regarding the ambiguity task):

 River Money Bank  [X].

Distractors were arranged by using one or two distractors on position one or two:

 River Wind Bank  [ ].

or

 Door Wind Bank  [ ].

Example (regarding the hyperonymy/hyponomy task).

 Water Juice Drink   [X].

Additionally, three classical adages were tested by giving three answer alternatives for each wording. HOLF has not been published before, and this is the first attempt to test the effect of high order language tasks with schizophrenic patients using a very simple design. HOLF uses German language items and has not been translated to the English language until now. HOLF is in an early stage of development, and further data concerning validity and reliability has to be collected in future studies. 

## 4. Statistic Analysis

Anonymised data was analysed with SPSS 15.0 for Windows (Statistical Package for the Social Sciences, Lead Technologies, Inc., Chicago, 2006). HOLF was analysed descriptively with regard to the number of items that were processed adequately. The sum score of correct answers were calculated on total HOLF scale as well as on the five subscales antonyms, homonyms, hyperonyms, synonyms, and adages. Since the distribution of raw scores was highly skewed with a large proportion of subjects delivering high sum scores, nonparametric tests (Mann-Whitney *U* test, Wilcoxon rank order test) were performed in order to compare the two subsamples (clinical population and healthy controls). 

## 5. Results

The sample comprised 40 patients diagnosed with schizophrenia according to ICD-10, F20, and 40 healthy controls. There were no significant differences in age and gender. The clinical subsample had a history of 4.1 previous hospitalisations on average and a severity-of-illness-score of 4.4 (moderately ill) according to Clinical Global Impression Scale (CGI) at the time of the investigation.

Statistical measurements of the HOLF scale and subscales as well as CLANG and PANSS are shown in Table 1. The internal consistency measured by Cronbach's alpha of HOLF total scale and subscales was high.

Healthy controls answered the items on HOLF and CLANG mostly without mistakes, whereas patients had significant problems with the task. The differences between the two groups on almost all subscales were highly significant with the exception if the adages scale.

HOLF total scale showed a significant correlation with CLANG even if controlled for psychopathology as measured by PANSS (*r* = −0.396, *P* < 0.001, df = 76). HOLF subscales correlated significantly with CLANG with the exception of adages scale. Correlation coefficients are shown in Table 2.

## 6. Discussion

The aim of the current study was to explore in how far schizophrenic symptoms are correlated with difficulties in high order linguistic processing. By testing high order language performance using the HOLF battery we found that schizophrenic patients showed significant difficulties in discriminating ambiguities, hypero- and hyponymy, and synonymy compared to healthy controls. Antonyms and adages showed less significant results comparing both groups. The group differences observed for antonyms and adages were weaker than the effects obtained for the other types of linguistic ambiguities. If this pattern is determined to be reliable, these latter linguistic features may have less pathognomonic value for schizophrenia. The more difficult a linguistic operation was the more confusion was measured within the schizophrenic group while healthy controls did not show significant problems solving high order language tasks. Age correlated slightly with general performance in HOLF, but there were more significant effects regarding the severity of illness and times of hospitalization. One might assume that age and times of hospitalization correlate with the chronicity of illness.

HOLF highly correlated with the standard instrument for scoring symptom load in schizophrenia called PANSS. We conclude that disturbed (high order) language function might be pathognomonic for schizophrenia. As already mentioned by Bleuler, “loosening of association” might be the core symptom regarding psychotic syndromes. Focussing on the correct word alternative while discriminating ambiguous meanings seems to be deeply disturbed in our patient group. According to our data, homonymy detection was highly impaired in patients with schizophrenia. 

In further studies, patients with schizophrenia showed problems in selecting context-related ambiguous meanings. They have been shown to be impaired in using the context of sentences to determine an appropriate meaning of a homographic word [29, 30]. Cohen and Servan-Schreiber [31] showed that schizophrenic patients have difficulties in processing multimeaning words, but only when disambiguating context precedes the target homograph. Previous research has suggested that a failure in processing contextual information may account for the heterogenous clinical manifestations and cognitive impairments observed in schizophrenia. Our data suggests difficulties of schizophrenic patients in processing multimeaning words such as ambiguities and synonyms but also suggests difficulties in solving semantic taxonomies such as hypero- and hyponymies. Antonyms were not presented in a randomised order but in a block design with several distractors. The antonym task within our experiment might have been too simple to solve, so these items did not show any differences between patient and control group. Therefore, we used the antonym task as a warm-up and to motivate patients to carry on with the more difficult tasks.

CLANG [32] was a first and important step in reactivating the Bleulerian idea of underlying language disturbance in schizophrenia but seems to be much too unspecific for detecting high order language dysfunction. CLANG has to be rated by the examiner who is always subjectively biased by the personal impressions and diagnostic data concerning his patient. HOLF now offers a more objective method which is highly significantly correlated with CLANG and PANSS. PANSS indeed is a well known and established diagnostic tool regarding schizophrenic symptoms. 

Delusions can be considered as deviations in the capacity to attach significance to the phonological representations that are the primary building blocks of words. Disruptions such as clause boundaries or sentence endings occur generally, particularly at points of attentional focus fluctuation [11, 33]. As a consequence, coherence of speech breaks down, which is fundamental for the development of formal thought disorder. According to our data one might assume that the more vague or ambiguous a semantic correlation is presented, the more effort has to be made by the neural system in processing these items. 

According to connectionist network models [34], each component of an utterance activates associated semantic units which remain activated for a finite period of time [35]. In schizophrenia, the speed of decay which is following the spread of activation seems prolonged. If such an inhibitory process becomes impaired, patients have a greater potential for intrusion into later thought and speech. Although context information is important for almost all cognitive tasks, the domain of language is ideally suited for the study of contextual processing. Homonyms and synonyms highly depend on correct contextual priming, so deficits in processing these features lead to massive linguistic irritation in the schizophrenic brain as seen in our experiment. 

Titone et al. [36] used a priming task by presenting sentences containing homonyms. Eighteen schizophrenic patients were asked on lexical decisions about visual targets related to the homonyms' subordinate, respectively, dominant meaning. When sentences biased subordinate meaning, patients showed priming of dominant targets. The results also suggest that contextual strength is an important determinant of when schizophrenia patients fail to inhibit contextually irrelevant meanings. Wentura et al. [37] investigated priming by using a masked repetition task and compared formal thought-disordered patients, nonthought disordered patients, and healthy controls. For thought-disordered patients they found “hyperpriming,” whereas the other groups showed regular priming. This result yields evidence for a lack of inhibitory function in thought- (and therefore language-) disordered patients. Furthermore, Sitnikova et al. [38] found that the N400 ERP component that is known to be sensitive to contextual effects was attenuated in patients with schizophrenia. This is potentially due to inadequate contextual suppression mechanisms and/or due to increased levels of word-meaning activation.

“Hyperpriming” was detected in our data too while there were many false-positive errors in the schizophrenia group. As already mentioned above, Salisbury [9, 10] described a model of initial hyper-priming by using ERP data investigating patients with schizophrenia. These findings suggest a neural correlate for the difficulties in suppressing correct word alternatives. 

In summary, using the HOLF test battery we found that schizophrenic patients showed significant difficulties in discriminating ambiguities, hypero- and hyponymy, or synonymy compared to healthy controls. Antonyms and adages showed rather significant results in comparing both groups, so these features seem to have a less specific pathognomonic and diagnostic value regarding schizophrenia or were too simple to solve. The more difficult a linguistic operation was, the more confusion was measured in the schizophrenic group while healthy controls did not show significant problems in solving higher order language tasks. 

One limitation of our study might be a very low error rate in healthy individuals. Therefore, very few errors cause a highly significant difference in performance between both groups. On the other hand, HOLF was able to detect schizophrenic symptoms on a very subtle level which might be an indicator for underlying language problems, although the patient group presented a low level of symptoms, and all patients have been well medicated. By revitalising the Bleulerian focus on thought, respectively, language disorder the almost chaotic mechanisms of psychotic experience might become more understandable. Our findings might inspire the development of early detection test batteries which include high order language functions in order to detect subtle and underlying language deficits in schizophrenic patients. HOLF is in a very early stage of development, and more data is needed especially regarding psychometric properties such as test-retest reliability and validity.

## Supplementary Material

This supplementary material is a pilot test called HOLF developed by our group. The first task was a warm up task consisting of 20 antonym relation pairs, while half of them were distractors. The second task represented other, mixed high order linguistic features, including synonymy, homonymy and hyperonomy vs. hyponomy. Each item group included 20 items while 10 of them were correct and 10 of them were distractors. Individuals were instructed by the examiner: “Please mark if the first words correlate with the last word in the line”.

## Figures and Tables

**Table 1:** Statistics of HOLF, CLANG, and PANSS by subsample (patients *N* = 40; controls *N* = 40).

Scale	Sample	Mean	SD	Median	Mean rank	Rank sum	Mann-*W U *	*Z*	*P*	Alpha
HOLF	Patients	68.5	13.6	72.5	21.5	860	39.5	−7.4	<0.001	0.963
	Controls	82.3	0.8	82.5	59.5	2380				
CLANG	Patients	0.4	0.4	0.2	57.5	2300	120	−7.3	<0.001	0.893
	Controls	0.0	0.0	0	23.5	940				
PANSS	Patients	1.5	0.4	1.4
	Controls	1.0	0.0	1
HOLF subscales										
Antonyms	Patients	17.9	3.7	19	30.4	1215	395	−5.0	<0.001	0.924
	Controls	19.9	0.2	20	50.6	2025				
Homonyms	Patients	13.9	4.1	14	22.3	891	71	−7.1	<0.001	0.878
	Controls	19.4	0.8	20	58.7	2349				
Synonyms	Patients	17.0	3.1	18	23.5	941	121	−7.1	<0.001	0.845
	Controls	19.9	0.3	20	57.5	2299				
Hyperonyms	Patients	17.0	4.5	19	28.1	1124	304	−5.6	<0.001	0.934
	Controls	19.9	0.2	20	52.9	2116				
Adages	Patients	2.8	0.7	3	38.5	1540	720	−2.0	0.042	0.898
	Controls	3.0	0.0	3	42.5	1700				
Distractors	Patients	10.2	5.6	13	29.8	1192	372	−4.4	<0.001	0.962
	Controls	14.7	0.5	15	51.2	2048				

**Table 2:** Correlation coefficients (patients *N* = 40; controls *N* = 40).

Spearman-Rho		Correlations (*n* = 40)											
		Severity of illness	HOLF all	HOLF distractors	HOLF antonyms	HOLF homonyms	HOLF synonyms	HOLF hyperonyms	HOLF adages	CLANG	PANSS	Age	Number of hospitalisations
	Correlation coefficient	1.000	−.653**	−.540**	−.371*	−.539**	−.431**	−.438**	−.266	.447**	.447**	.475**	.508**
Severity of illness	Sig. (2 sides)	—	.000	.000	.018	.000	.006	.005	.097	.004	.004	.002	.001
	*N*	40	40	40	40	40	40	40	40	40	39	40	40

	Correlation coefficient	−.653**	1.000	.826**	.483**	.814**	.792**	.670**	.401*	−.696**	−.743**	−.338*	−.128
HOLF all	Sig. (2 sides)	.000	—	.000	.002	.000	.000	.000	.010	.000	.000	.033	.431
	*N*	40	40	40	40	40	40	40	40	40	39	40	40

	Correlation coefficient	−.540**	.826**	1.000	.378*	.488**	.762**	.703**	.300	−.598**	−.704**	−.241	−.101
HOLF distractors	Sig. (2 sides)	.000	.000	—	.016	.001	.000	.000	.060	.000	.000	.134	.534
	*N*	40	40	40	40	40	40	40	40	40	39	40	40

	Correlation coefficient	−.371*	.483**	.378*	1.000	.348*	.397*	.370*	.421**	−.474**	−.373*	−.268	−.439**
HOLF antonyms	Sig. (2 sides)	.018	.002	.016	—	.028	.011	.019	.007	.002	.019	.094	.005
	*N*	40	40	40	40	40	40	40	40	40	39	40	40
	Correlation coefficient	−.539**	.814**	.488**	.348*	1.000	.517**	.255	.474**	−.446**	−.446**	−.343*	−.089
HOLF homonyms	Sig. (2 sides)	.000	.000	.001	.028	—	.001	.112	.002	.004	.004	.030	.586
	*N*	40	40	40	40	40	40	40	40	40	39	40	40
	Correlation coefficient	−.431**	.792**	.762**	.397*	.517**	1.000	.592**	.401*	−.614**	−.766**	−.131	−.096
HOLF synonyms	Sig. (2 sides)	.006	.000	.000	.011	.001	—	.000	.010	.000	.000	.419	.556
	*N*	40	40	40	40	40	40	40	40	40	39	40	40

	Correlation coefficient	−.438**	.670**	.703**	.370*	.255	.592**	1.000	.198	−.674**	−.603**	−.166	−.090
HOLF hyperonyms	Sig. (2 sides)	.005	.000	.000	.019	.112	.000	—	.221	.000	.000	.305	.581
	*N*	40	40	40	40	40	40	40	40	40	39	40	40

	Correlation coefficient	−.266	.401*	.300	.421**	.474**	.401*	.198	1.000	−.310	−.180	−.180	−.297
HOLF adages	Sig. (2 sides)	.097	.010	.060	.007	.002	.010	.221	—	.051	.273	.266	.063
	*N*	40	40	40	40	40	40	40	40	40	39	40	40

	Correlation coefficient	.447**	−.696**	−.598**	−.474**	−.446**	−.614**	−.674**	−.310	1.000	.830**	295	.186
CLANG	Sig. (2 sides)	004	.000	.000	.002	.004	.000	.000	.051	—	.000	065	.251
	*N*	40	40	40	40	40	40	40	40	40	39	40	40

	Correlation coefficient	.447**	−.743**	−.704**	−.373*	−.446**	−.766**	−.603**	−.180	.830**	1.000	.077	−.011
PANSS	Sig. (2 sides)	.004	.000	.000	.019	.004	.000	.000	.273	.000	—	.641	.948
	*N*	39	39	39	39	39	39	39	39	39	39	39	39

	Correlation coefficient	.475**	−.338*	−.241	−.268	−.343*	−.131	−.166	−.180	.295	.077	1.000	.395*
Age	Sig. (2 sides)	.002	.033	.134	.094	.030	.419	.305	.266	.065	.641	—	.012
	*N*	40	40	40	40	40	40	40	40	40	39	40	40

**The correlation is significant on a 0.01 niveau (2 sides).

*The correlation is significant on a 0.05 niveau (2 sides).
