# Supplementary material for: High Order Linguistic Features Such as Ambiguity Processing as Relevant Diagnostic Markers for Schizophrenia
Source: Schizophr Res Treatment. 2012 Dec 11;2012:825050. doi: 10.1155/2012/825050 (PMC3529898; doi:10.1155/2012/825050)
Supplement: Supplementary file 1 — This supplementary material is a pilot test called HOLF developed by our group. The first task was a warm up task consisting of 20 antonym relation pairs, while half of them were distractors. The second task represented other, mixed high order linguistic features, including synonymy, homonymy and hyperonomy vs. hyponomy. Each item group included 20 items while 10 of them were correct and 10 of them were distractors. Individuals were instructed by the examiner: “Please mark if the first words correlate with the last word in the line”. [file 825050.f1.docx]

**Attachment:**

HOLF - Test

**KlientIn ID ⎣⎦⎣⎦-⎣⎦⎣⎦⎣⎦ Arzt/Ärztin ID ⎣⎦⎣⎦⎣⎦**

**Datum ⎣⎦⎣⎦ ⎣⎦⎣⎦ ⎣⎦⎣⎦**

**Tag Monat Jahr**

Im Folgenden finden Sie jeweils zwei oder drei Wörter in einer Reihe. Bei einigen der Wortpaarungen besteht eine Beziehung des ersten bzw. der ersten beiden Wörter zum letzten Wort. *Wenn Sie eine solche Beziehung erkennen, kreuzen Sie bitte das freie Feld an.*

Vielen herzlichen Dank für Ihre Unterstützung.

ERKENNEN SIE EINEN ZUSAMMENHANG? WENN JA, BITTE ANKREUZEN.

heiss kalt [ ]

spitz stumpf [ ]

rauh glatt [ ]

hell eckig [ ]

schön hässlich [ ]

endlos begrenzt [ ]

roh schwach [ ]

heiss klug [ ]

spitz ruhig [ ]

rot trocken [ ]

fettig glatt [ ]

tot lebendig [ ]

nass trocken [ ]

hell dunkel [ ]

rund lebendig [ ]

aggressiv friedlich [ ]

warm hässlich [ ]

mutig friedlich [ ]

endlos kantig [ ]

stark schwach [ ]

ERKENNEN SIE EINEN ZUSAMMENHANG DER ERSTEN BEIDEN WOERTER ZUM LETZTEN? WENN JA, BITTE ANKREUZEN.

Kind Schraube Mutter [ ]

Baum Knochen Kiefer [ ]

Aufwendungen Kerne Unkosten [ ]

Strasse Wand Kiefer [ ]

Lenkrad Abgabe Steuer [ ]

Brille Mond Pflanze [ ]

Locher Cobra Schlange [ ]

Musik Vogel Flügel [ ]

Gegenstand Ding Objekt [ ]

Hut Motor Zylinder [ ]

anbieten telefonieren Feld räumen [ ]

Apfel Heft Steuer [ ]

Gemüse Optik Linse [ ]

Hut Mantel Kleidung [ ]

Geschirr Hülse Schale [ ]

weichen abziehen Feld räumen [ ]

Gewicht Gefäss Tonne [ ]

Material Hut Zylinder [ ]

Tanne Buche Baum [ ]

Grenze Länge Zoll [ ]

Sohn Gardine Schale [ ]

Bazillus Keim Bakterium [ ]

Kasette Himmel Getränk [ ]

Haus Tasche Flügel [ ]

erbitten beantragen Gesuch stellen [ ]

Grenze Telefon Zoll [ ]

Gangster Dieb Ganove [ ]

Rente Himmel Nagel [ ]

Mars Venus Planet [ ]

Hand Sonne auf der Hut [ ]

Auto Schraube Mutter [ ]

Ente Haus Planet [ ]

Apfel Schraube Obst [ ]

Blatt Gewicht Tonne [ ]

Wasser Cola Getränk [ ]

Auto Buche Baum [ ]

Leber Taxi Organ [ ]

Apfel Buch Ebene [ ]

Blume Baum Pflanze [ ]

irrtümlich nicht mit Absicht aus Versehen [ ]

Stempel Boot Kleidung [ ]

Vater Gans Möbel [ ]

Katze Pferd Tier [ ]

Tisch Schrank Möbel [ ]

Leber Herz Organ [ ]

Finger Hammer Nagel [ ]

Katze Quittung Tier [ ]

Flachland Plateau Ebene [ ]

beantragen reiten Gesuch stellen [ ]

Aufwendungen Ausgaben Unkosten [ ]

Ding Vater Objekt [ ]

misstrauisch wachsam auf der Hut [ ]

Dieb Amsel Ganove [ ]

Natter Cobra Schlange [ ]

irrtümlich grün aus Versehen [ ]

falten brennen Ziel erreichen [ ]

Apfel Traube Obst [ ]

Optik Griff Linse [ ]

ausschalten schreiben Bakterium [ ]

ankommen gelingen Ziel erreichen [ ]

WAS BEDEUTET DAS SPRICHWORT: “WER ANDEREN EINE GRUBE GRÄBT, FÄLLT SELBST HINEIN”? ZUTREFFENDES BITTE ANKREUZEN (A-C).

(A) Man sollte aufpassen, dass man beim Arbeiten nicht in seine eigene Grube fällt.

(B) Jemand, der einem anderen Schaden zufügen möchte, läuft Gefahr, am Ende selbst der

Geschädigte zu sein.

(C) Wenn man für jemanden eine Grube gräbt, sollte man schauen, dass man dabei nicht

ausgenutzt wird.

WAS BEDEUTET DAS SPRICHWORT: “DER APFEL FÄLLT NICHT WEIT VOM STAMM”? ZUTREFFENDES BITTE ANKREUZEN (A-C).

(A) Charaktereigenschaften z.B. eines Vaters finden sich oft auch bei dessen Kindern.

(B) Wenn ein reifer Apfel vom Baum fällt, liegt er zumeist unmittelbar daneben.

(C) Bei der Apfelernte sollte man sich vor herunterfallenden Äpfeln in Acht nehmen.

WAS BEDEUTET DAS SPRICHWORT: “ZU VIELE KÖCHE VERDERBEN DEN BREI”? ZUTREFFENDES BITTE ANKREUZEN (A-C).

(A) Beim Kochen sollte man aufpassen, das nicht fremde Köche das Gericht verderben.

(B) Wenn viele Köche ein Gericht kochen, ist das Ergebnis oft ein einziger Brei.

(C) Wenn zu viele Menschen an einer Aufgabe arbeiten, schadet das oftmals eher dem Endergebnis als das es nützt.
